# Working collaboratively with an online advisory group of people with learning disabilities in covid-times: carrier pigeons, cats and drones

**DOI:** 10.1186/s40900-023-00494-7

**Published:** 2023-09-09

**Authors:** Eppie Leishman, Deborah Quilgars, David Abbott, Sam Clark, Becca Cooper, Andy Pollin, Stephen Hodgkins, Paul Scarrott

**Affiliations:** 1 University of York, York, UK; 2 University of Bristol, Bristol, UK; 3 Learning Disability England, Birmingham, UK; 4 York People First, York, UK; 5 My Life My Choice, Oxford, UK

**Keywords:** Learning disabilities, Advisory group, Online research, Inclusive research

## Abstract

While much attention and emphasis have been given to the role and value of advisory groups in social science research, less has been published on the experiences of those involved in such collaborative efforts. This article reflects on the experiences of academics, collaborators and self-advocacy experts who formed an advisory group for a research project focused on people with learning disabilities’ experiences of renting their own homes. Our paper describes the collaboration, how it changed because of Covid and because of changing relationships, and what worked well and what was challenging. This is in part because these more transparent accounts of working together are sometimes missing from research. We discuss issues relating to bureaucratic research systems which are largely inaccessible to people with learning disabilities and how we approached these. We also highlight the joys and benefits of the research approach that we adopted as well as the challenging and more difficult aspects.

## Abstract (plain language summary)

This article tells the story of a research project about people with learning disabilities who rent their own homes in England. The article is not so much about the research findings but more about how the research team worked together. This group included self-advocacy experts with learning disabilities, research collaborators and academic researchers. At the start of the project, people with learning disabilities were invited to be part of an advisory group. But as the project went on, this group started to challenge the limits of the role and wanted to be more involved. This changed the course of the research—as well as the fact that it was happening at the same time as the Covid pandemic. The group had some difficult issues to deal with including complicated ethics processes, bureaucracy about getting people paid, and disagreements about language and terminology. We had some hard conversations about the words we use in academia and in real life and who gets to do research. We also had lots of fun wearing silly glasses at Christmas and talking about carrier pigeons. In the end we all felt that the way the research was carried out had improved the project overall.

## Introduction

This paper outlines the experiences of the ‘renting your own place’ research team. We are a team of academic researchers, collaborators and self-advocates (people with learning disabilities who advocate for their own rights, often as part of a self-advocacy group) in England who worked together in 2020–23 on research about people with learning disabilities who rent their own homes in the private and social housing sectors. There is more information about the study at https://www.york.ac.uk/chp/housing-health-wellbeing/learning-disabilities/. Some of us writing this paper have learning disabilities and some of us do not. We want to use this paper to tell other people about our experiences, celebrate the good things we have been able to achieve, and share some of the more difficult aspects of the research process. Below, there is a photo of some of us working together at the University of York.
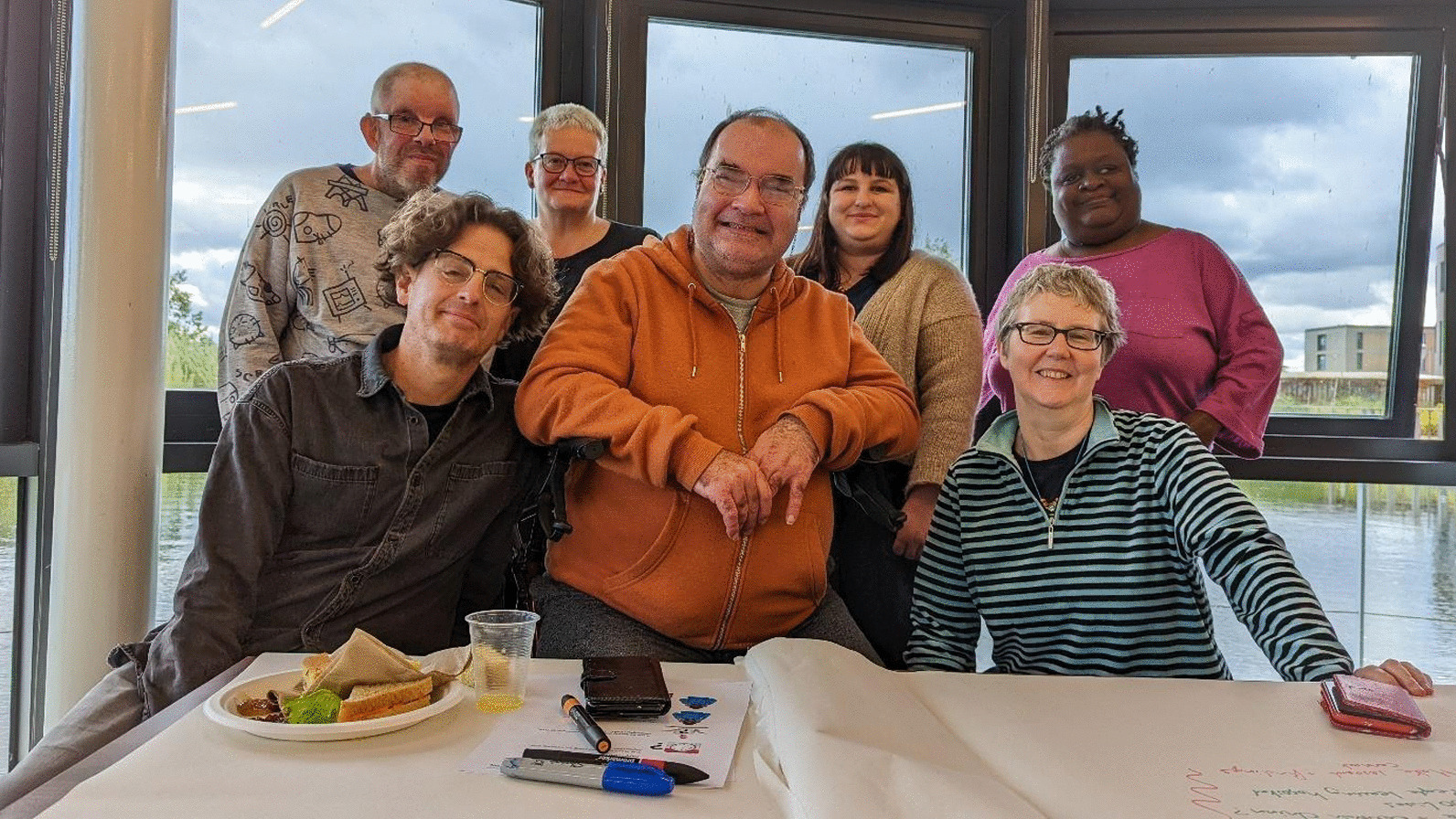


While there is a longstanding tradition of transparency in qualitative research [1], the practicalities, experiences and impacts of collaborative working can sometimes remain a footnote in the story of a research study. We would like to contribute to the conversations started by others [2] on including people with learning disabilities in research through online methods, by discussing the practicalities, challenges and joys of working together during a global pandemic.

In this paper, we will start by thinking about what others have written about inclusionary ways of doing research. The paper will then tell the story of our research and talk about who was involved in our research team and what we did. We hope it might help people who are thinking of doing something similar. We will then discuss what went well on the project, how our approach may have made our research better, the things that were more challenging, and reflect on what could have gone better.

This paper and our project were often troubled by terminology—academics, collaborators, self-advocacy experts, co-researchers. Power et al. [3] reflect on the power and politics of these labels and the danger of jargon, whilst Black et al. [4] write about ‘experts by experience’ to sit alongside the academic training that gives academics expertise in research. This gives everyone the label of expert—although this does not necessarily mean that power relations are equalised by the orchestration of language. In this paper we describe ourselves as: academics/academic researchers—University based researchers at two English universities (Leishman, Quilgars and Abbott); research collaborators—Learning Disability England, a user led organisation of people with learning disabilities (Sammantha Clark); Stephen Hodgkins, an artist, a support worker and an activist; self-advocacy experts—people with learning disabilities who have lived experience of the issue being researched (they all rent their own homes) and who are part of the self-advocacy movement (Cooper, Pollin and Scarrott).

## Designing this paper

As a group we have discussed this paper many times. We have agreed that everyone involved in the group should be an author. We have attempted to minimise the amount of academic jargon but recognise that the paper would, in its entirety, not be wholly accessible for people with learning disabilities. The impetus for the paper came from the academics working on the project who were keen to write about the processes of inclusion and collaboration, something we had found useful when we had seen others do it [5, 6]. Given the level of co-researching that emerged in our research, it was vital that there was a whole-team dialogue about this paper in terms of content and authorship.

The academics on the project asked the research collaborators and self-advocacy experts to lead an on-line session with a focus on what it had been like to work together. In collaboration with self-advocacy experts, the research collaborator devised a series of questions for the group to reflect on to help shape and inform this paper. In the end these conversations continued formally and informally over several months. Although the contents of this paper were discussed at various points, the act of writing itself was largely undertaken by the academic researchers, although there are quotes from the self-advocacy experts throughout the paper. Self-advocacy experts were happy to be named as co-authors as long as an easy read of this paper was produced, and that a hard copy of any article published was sent to them so that it could be shared with family and friends. When we received reviewer comments we had 1–1 phone conversations between the academic researchers and the self-advocacy experts to talk about the issues raised.

## Background

While there have been many ways of approaching the inclusion of members of the public, people with lived experience, and the users of health and social care services in research, *inclusive research* has been argued to be a relatively accessible term in the important distinctions between emancipatory, participatory and coproduced terminology [7]. In regard to learning disability research, inclusive research has been described as research in which people with learning disabilities play an active role in the research process and are not just the subjects of the research [8–10]. This has not always been the case in learning disability research which has a history of researchers focusing ‘on’ rather than ‘with’ people with learning disabilities [11–13]. Self-advocates and self-advocacy organisations, including the People First movement, have been rightfully acknowledged for their significant role in challenging and reshaping these norms of research practice and the production of knowledge [14, 15].

In the last two decades, increasing emphasis has been placed on the meaningful engagement of the public in health and social care research [16, 17] and is now encouraged or required by organisations that fund research. For transparency, this is the case in our research which was funded by the National Institute for Health and Care Research, School for Social Care Research (NIHR SSCR) which has long emphasised the importance of public involvement in its funded research projects. We also know that debates about who is involved in research are contentious. Well established narratives include whether inclusion in research is tokenistic; the real differences between participatory research and research which is organised more radically in terms of how power is organised; the co-opting of terms/language around coproduction in particular; the lack of clarity and interchangeable use of terms to describe what actually happens when different people collaborate in research.

Furthermore, the external pressures faced by university researchers have previously been highlighted as a challenge to collaborative research. For example, academic researchers’ performance and job requirements emphasise the value of first authored papers in ‘good’ peer-reviewed academic journals [15, 18]. Academic researchers often work to tight deadlines, balancing multiple roles, and their corresponding requirements and academic writing practices can be highly exclusionary. Squeezing in writing time around other tasks is not always conducive to collaborative efforts. In the same vein, collaborators with lived experience and/or user led organisations may or may not be interested in writing for journal articles and also have busy lives. That said, co-writing and co-authorship between academic researchers and researchers with learning disabilities is not at all new (Val Williams at the University of Bristol was an early proponent publishing as a team comprised of academics and self-advocates [19]) and the *British Journal of Learning Disabilities* and the *International Journal of Disability and Social Justice* both routinely include either easy-read or plain language summaries of articles in their journals [20].

The details of who we are and what we did and how we worked together follow. Firstly, it is worth noting that our mutually agreed description of how we worked together is not a neat one. In part because things changed more than once in how we worked together and because in ways that nobody could have anticipated, we found ourselves working together on-line because of the Covid pandemic. The projects inception was one of collaboration in that academics did not come up with the idea in isolation. The topic was gaining traction in policy and practice circles and conversations began between academics with an interest in the intersections of housing, social care and learning disability, and organisations of and for people with learning disabilities, who also thought that more research was needed about housing. A user-led organisation of people with learning disabilities formally partnered with university researchers in a bid submitted for funding which envisaged collaborative working i.e. working together on aspects of the research but with academic researchers doing most of the ‘traditional’ aspects of the research, notably, interviews and data analysis. The partnership also included two national housing associations who, for reasons we will explain, did not play an active role, and a community activist and artist who led on creative and visual aspects of our research. The degree of collaboration became contentious and as a result changed as the project progressed such that aspects of the study could be described as co-designed. Whether any aspect of the project could truly be described as coproduced is arguable given that the academics remained in charge of the project budget and were ultimately accountable to the research funder. We explore these tensions below.

## What we did and how we worked together

Our research was funded to explore the experiences of people with learning disabilities who rent their own homes from either a private landlord or social housing provider in England. We were particularly interested in understanding what support people were or were not getting to rent. We planned to interview some people who got small amounts of statutory social care support and some people who did not. The research comprised a search of English local authority housing strategy documents to look for the inclusion or absence of specific discussion of housing for people with learning disabilities; a series of on-line regional workshops attended by over 100 people for anyone interested in the specific issue of people with learning disabilities renting their own homes; 35 qualitative interviews with people who were renting their own homes; and the use of creative booklets for people to capture words, images and photographs that communicated things about how they felt about their home.

When this research was planned and funding given, we thought we would be able to do the qualitative interview component of the research in person i.e. go to people’s homes and speak to them about what it was like living there, and also ask them to help us by taking part in a follow up strand of work with a more creative bent which we planned to involve an advisory group of people with learning disabilities in more centrally. Our advisory group of nine people with learning disabilities was set up to give us overall guidance on our research at 3-monthly intervals in person. Alongside this, was a wider steering group which included policy makers, housing providers, commissioners and other housing academics. However, once the research began in September 2020 amid the Covid pandemic, we had to radically rethink our approach and not least because delays and inaction in seeing people with learning disabilities as a high-risk priority group were to place them at greater risk from Covid [21]. In addition the two housing organisations named on our bid and included in large part to help with recruitment both signalled that they would not be able to take part as planned due to service pressures.

Several challenges presented themselves to us almost all at once: how to interview people from a distance instead of in person; how to explain to and support people who wanted to take part in the creative part of our research (which involved them having a camera and an arts book to record images and less word-based information about how they felt about renting their own homes) when we would not be able to do this face to face; how to work with an advisory group of people with learning disabilities: what technology would we use, who would have access to it and who might not, would it be reliable, would people still want to take part given the enormity of living in Covid-times [22].

We knew that at the time of forming the advisory group that public involvement in research was facing significant challenges due to the Covid-19 pandemic. Anecdotal concerns from the time were evidenced by later findings from the Health Research Authority which experienced a rapid decline in the number of ethics applications including public involvement [23, 24]. The pandemic meant we needed to change our plans but also gave us different ways of thinking and working. The biggest change was that all our meetings needed to happen online. The researchers and collaborators had concerns about the digital inclusion of people with learning disabilities and over who may be left behind by the sudden switch to online working [25].

Because of our collaboration with Learning Disability England we had indications that more people with learning disabilities were using technology, like Zoom than before the pandemic, and while concerns over exclusion remained, this gave us an opportunity to include people in our research process who would not be able to help if they had to come to lots of meetings at the main base for the research, the University of York. The advisory group had an initial membership of nine: the three academic researchers working on the project who had overall responsibility for the project and would undertake the interviews, two research collaborators who both worked with and in self-advocacy organisations, and four self-advocacy experts who have learning disabilities and rent their own homes, three of whom were happy to be named as co-authors on this paper.

The self-advocacy experts with learning disabilities who rented their own homes were recruited by the research collaborators. This approach meant that the experts recruited had a prior connection and familiarity with at least one other member of the advisory group. These familiar intermediaries could therefore be drawn upon to ensure any access needs were met and offered a point of contact outside of the group should any issues arise. We felt this was particularly important as all contact was happening at a distance (on the phone or online) in a time of huge uncertainty for all those involved. In a similar fashion to that suggested by others [26], research collaborators acted as a bridge between the self-advocacy experts and the academic researchers who had different levels of experience of working with people with learning disabilities and indeed of undertaking inclusive research. As Staniszewska et al. [27] point out, high quality relationships build trust, and we were able to utilise existing relationships to form new ones.

Meetings of the advisory group were held across the duration of the project. The self-advocacy experts told the academic researchers and collaborators in the first meeting that they wanted to be included regularly in the project in online meetings and not by email or other ways of updating people. The group also challenged the researchers when they felt it had been too long since we had all met. It was an early indication that the group did not want to swing into action at the academic’s beck and call but wanted to be much more systematically and regularly involved. In fact it was quite clear that the self-advocates did not want to be an advisory group in the traditional sense of the term at all i.e. a relatively passive group with a focus on consultation rather than participation and which might meet on only an occasional basis. They wanted to play a much fuller part in the study and on an equal footing. All online meetings took place using Zoom software which was a strong preference of the self-advocacy experts who were familiar with Zoom. After the first meeting, we established, for accessibility reasons, the easiest way to format the meeting was to have no passcode and to use the same Zoom link for all subsequent meetings. This link was included in all communication about the meetings. We were very conscious of Zoom fatigue and the nature of online meetings and so they were scheduled to last one hour with one exception of a Christmas meeting followed by a social which lasted for two hours. 

The first author took responsibility for the administration of the meetings and took certain steps to support the meeting, including logging on to every meeting around ten minutes early; emailing reminders on the day of every meeting; and being the person responsible for dealing with all aspects of paying people. Although initial meetings had an easy-read agenda prepared in advance, we moved away from this when it felt that the academics were doing the agenda-setting.

It was important that everyone was paid properly. The self-advocacy experts were offered personal payment or payments to their organisations. All but one took payment, one self-advocacy expert preferred to take on the role in an unpaid voluntary capacity. Payment was made at Involve rates [28]. Research collaborators were named collaborators on the funding awarded and were therefore paid for their involvement in the research as were the academic researchers. Paying self-advocacy experts proved to be complicated and took lots of time. University systems were bureaucratic and inaccessible. We settled on making lump sum, honorarium payments to our self-advocacy organisations who were better equipped to respond flexibly and accessibly to people’s individual financial needs, constraints and wants. So whilst we found ways to make payments work, we did not feel supported or enabled by the University to do this despite it being an essential part of our research funding [29].

## Working together on research tools

One of our first tasks was to create the main tools for data collection—in this case qualitative interviews. We talked a lot about the experiences of the self-advocacy experts and used these experiences to think about the sorts of things we needed to ask our participants. What worked well was not just discussing this once but returning to the discussion over several meetings with the academics refining the ideas in between. The themes we agreed upon to shape our actual questions included: finding your own place, what makes a home, information, support, community, lockdown, the future, and renting advice for other people with learning disabilities. These themes were added to by the researcher collaborators who were also interested in policy both locally and nationally.

When the academic researchers drafted the project information sheet which told people about our research, an initial draft included photographs of the three researchers with their front doors, and the logos of the research collaborators and funder. This was strongly challenged by the self-advocacy experts who expressed feelings of being left out. They thought it important to include their photographs and self-advocacy group logos. They argued that if these were not included then people thinking about taking part would not be reassured that people with learning disabilities were actively involved in the research team. The academic researchers had clearly got this wrong and the information sheet was changed.

The information sheets and consent forms presented other challenges in terms of our inclusive approach. We discussed in depth what we thought renters needed to know about our research in order to reach an informed decision on taking part. We produced easy-read information sheets and consent forms informed by self-advocacy experts, collaborators with expertise in easy-read, and researchers with decades of research experience. However, the internal University approval process expanded the documents significantly by adding, for example, inaccessible and quite lengthy information about, for example, data protection, data storage and research governance. The project information sheet was 15 pages long while the consent form was another 5 pages with 17 ‘yes’ or ‘no’ statements! Though we made some changes we had to largely accept the additions to proceed. Informed consent and data protection are of course key principles of ethical research, however, in the end the documents did not reflect the group’s wishes. In fact one participant thought that the interview was finished when they had only reached the end of the consent form.

Working on research tools brought an early challenge from self-advocacy experts about who was going to do the research interviews. The research design was that the interviews would be undertaken by the academic researchers but the self-advocacy experts who had co-designed the research tools including interview questions, raised concerns about why they were not undertaking the interviews themselves. “I would have liked to have got out to people with learning disabilities, not just the academics,” said one self-advocacy expert. Another self-advocacy expert also felt that they might have been better at asking questions in plainer, more accessible ways and would have favoured doing the interviews in pairs with one academic researcher and one self-advocacy expert:

“Would love to go into the places they are livingand talk to the people face to face and get a live feeling of it. I would love to do that because I know you guys are like the professional side and we are taking what you heard from the interview, but we talk to the person who live in that place and maybe you ask questions, and they might not understand it, but we make it simplified.”

The academic researchers asserted that it was too late to make such a fundamental change to the project and acknowledged that it could and should have been done differently. This was arguably a weak response to a legitimate challenge and that weakness was not lost on the self-advocacy experts, one of whom commented:

“I don’t know how much funding you have for that project... we could have said to the funders we want a little bit extra because this is what we want to do.”

It may well have been very possible to negotiate more time and money from the funder to accommodate such a change but that did not happen. Why? The researchers acknowledge, with hindsight, that they lacked the confidence to deviate too far from the original plan, and were worried about going over time, over budget and over allocated workload if changes were made.

## Ethics

How to meaningfully include the advisory group in the research ethics process was a significant concern for the academic researchers on the project. One of the requirements of our research funder was that we needed to go through the Health Research Authority (HRA) ethics approval process. This process starts with a long online form that the academics had to complete, and the researchers struggled to find a way to include the advisory group in an accessible way. However, underpinning the final ethics application were all the co-designed research tools. Once submitted, we were invited to attend an ethics committee panel. These panel meetings can sometimes be daunting and difficult [30], and the academic researchers decided that the lead academic and one of the collaborators that represented self-advocacy groups should attend. This was not openly discussed with the self-advocacy experts. The academics were worried that the meeting would be run inaccessibly and based on experience, sometimes abrasively [31]. But this was an act of inappropriate protectionism even though, as predicted, the meeting *was* both challenging and technical. The self-advocacy experts said, when we discussed this after the event, that the meeting would have held no fear for them, and had specific suggestions for how it could have been handled better:

“A group of us – we would have taken the floor! Or the panel should come out and ask us questions.”

“Yeah I would like to come because what it was yeah... you’ve got someone with lived the experience... might give them more courage about why we’ve wanted to do it. Better to have a representative to support you and you supporting us.”

In reflecting on how this process could be improved, we discussed how having people with learning disabilities on the HRA ethics approval panel would not only symbolically indicate inclusion but would encourage changes to the process itself. The self-advocacy experts also pointed to the inaccessible nature of the committee feedback and said that it should have been in, ‘…easy read with pictures.’

## Inclusion and exclusion

Issues of inclusion and exclusion often loom large in the everyday lives of people with learning disabilities. For the academic researchers the idea of inclusion and exclusion criteria for the research was normal and relatively uncontentious. Self-advocates were reluctant to exclude anyone with a learning disability who was renting their place in any circumstance i.e. with family or with a shared care scheme. They took the issue of ‘exclusion criteria’ seriously and personally. The academic researchers referenced the terms of the funding agreement and the limits of what could be done but it was an issue that remained a source of unease, and looking back at this topic, the self-advocacy experts made a number of comments:

“It was quite harsh because we know people who moved back with their families.”

“I think everybody should be included...and I think it should be everyone buying their own house or renting their own house.”

The self-advocacy experts were sensitive to areas of potential exclusion throughout the project. When deciding how to do research at a distance, concerns over access to the internet, technology and overall accessibility of the project were discussed in depth. How would we enable someone who was blind or deaf to share their experiences? How could people who might not have, or who might need support to use, the internet take part? How might privacy and freedom to speak about personal things be affected if another person needed to be present? We built flexibility into our approach to account for this. Participants could do an interview using their chosen method—Zoom, Microsoft Teams, Facetime, Skype, Google Meet—or indeed take part on the phone. We did not always have official institutional support to offer this full range of tech options, but we did so anyway because otherwise we would have had only very small numbers of respondents and because it seemed disrespectful to mandate *our* preference.

One Black self-advocacy expert reminded the group that everybody else in the team was White and that a lot of research about people with learning disabilities focuses upon White people [32]. They said they would have especially liked to interview Black people with learning disabilities:

“Actually ask people from Black and ethnic minorities because they don’t get asked questions or if they do it’s not from somebody who knows what it’s like.”

Although the wider group would say that they shared these concerns, the push to not be complacent about the issue came from the group member affected. It should of course not be the responsibility of racially minoritized people to be the ones expected to call attention to this, not least because of the emotional labour involved, but this was indeed what was happening.

## Language and terminology

The research title reflected the academic preference for the term ‘learning disability’. Our main collaborator was an organisation called Learning Disability England and so there was a general assumption that this was the appropriate terminology. Some self-advocacy experts felt very differently and argued that the term was an antisocial model approach that situated disabling barriers within a person rather than without i.e. societal and structural ableism. These conversations and disagreements became far more than a discussion about language. They reflected long standing issues about whose view is foregrounded and privileged, and the wrongful assumption that all people with learning disabilities will think the same [33]. One of the academic researchers was adamant that the preferred term by consensus in the learning disability community was just that and that view prevailed even though the fundamental disagreement continued. Reflecting on the issue, the self-advocacy expert with the strongest views against the use of the term learning disabilities agreed that a team decision had been made, but said:

“It was quite debilitating. Me and [other team member] say learning difficulties but [other team member] says learning disabilities. We can kick up a debate but then go with the percentage. I think me and [other team member] couldn’t sort of say anything so we just went along with it”.

On the other hand, the self-advocacy expert who preferred the term ‘learning disability’ had equally strong views about it—“I think learning difficulties is a difficult meaning…it puts a downside on us.” The third self-advocacy expert thought that the most important thing was that everybody had had a chance to air their view—“It was really important to talk about it and ask people opinions.”

Similar contentious questions arose when the group talked about the labels of ‘mild’ or ‘moderate’ learning disabilities (the group of people described in the funding proposal as the groups of focus). Not all the self-advocacy experts were happy with the use of these terms which they felt were ‘medical model’ descriptors and not necessarily reflective of people’s experiences or self-understanding. While the academic researchers used the terminology to distinguish a group felt not to have been spoken with enough, particularly in terms of renting, others felt these were unnecessarily divisive terms. We resolved this by removing the terms ‘mild’ or ‘moderate’ from the research and instead agreed to use the more relevant (for this research) labels of people with learning disabilities with either no, or only small amounts of, statutory social care support.

We carried on with the term ‘advisory group’ throughout the project even after roles changed and evolved. It became the shorthand for what were in fact whole-team research meetings which became the default way that all of our discussions and decisions about the research were arranged.

## Data analysis

The academic researchers were mindful of the fact that public involvement in research does not *routinely* include data analysis. But we also knew of other studies that had involved people with learning disabilities in this work [11]. By the data analysis stage, the team was working much differently than at the start, in that there was no real need to think about whether to include or exclude anyone from data analysis, the only question was how to make it work. In practical terms, attempting the process on Zoom in an accessible format was very difficult. The self-advocacy experts wanted to listen to audio clips of interviews but finding anonymized sections which were not easily identifiable was tricky. (Of course this issue was linked to the fact that self-advocacy experts had not been included at an appropriate stage as people permitted to have access to the raw data.) The transcripts themselves were often very long (around 26 pages of typed text) and the stories people shared were not always easy to follow. While we did hold a one hour Zoom meeting to focus on themes relating to renting, listening to short audio clips without more context about the renters and their experiences did not generate a huge amount of discussion, or give the academic researchers a clear steer on what to look for in the data. We decided doing analysis online just for an hour at a time was not going to work. This obstacle coincided with the lifting of Covid restrictions as well as anappetite in the whole group to finally meet in person. We therefore planned our first in-person meeting after twenty months working on the project, in York in September 2022. We decided to have dinner together the evening before a full day focusing on analysis.

On that day, we listened to short clips about support with context about who these renters were. We covered tables in paper and wrote notes and drew pictures to show what we had been thinking about. The academic researchers noted their own tendency to try and manage time and tasks quite tightly to ‘get the work done’ which needed to be resisted. In splitting the day into two very broad sessions, one hearing audio clips and one looking at what people had included in their creative booklets, the self-advocacy experts began analysing what was going on within, behind and around the pictures and words. There was a shared ‘sense-making.’ A picture of a telephone led to discussions about who gets to answer a phone and who is listened to—a theme which became central to the analysis i.e. whose voices are heard?

## Relationships

There was a lot of joy and a lot of fun in the advisory group, and we suggest that this is not irrelevant or unimportant. Meetings were informal, chatty, distracted and often unguarded. There were running jokes about how best to find, recruit and contact people with suggestions including carrier pigeons, cats and drones—ideas became metaphors, became ritual and comedy and group bonding. They came from very early discussions of communication in the times of Covid and have stayed with us throughout. A group identity quickly developed, and we would, for example, check in on those unable to attend, send cards for bereavements and absences. We had social time together on-line and circulated Christmas boxes with treats including novelty glasses which were worn at an online party. At our first in-person meal we shared handmade presents of jewellery.

Perhaps this time building trust and relationship meant that the group could sustain difficult conversations and disagreements. While we were worried at the outset that developing rapport and dealing with tensions might be a challenge in an online space these fears did not play out. As a group geographically spread out, we were able to meet far more frequently than we would have in person. We also saw into each other's domestic spaces when the in-person norm is for a researcher to access the domestic space (usually home) of the researched person. Looking at, commenting on and laughing about all our home spaces, our pet interruptions, our taste in decoration was a more equalising frame than we had previously experienced.

## Discussion

In this section of the paper, we will discuss the overarching things that we believe we learnt as a group.

Relationships were key—a finding about doing research collaboratively that is not new. We quickly found that all team discussions and interactions mattered and were important. As the work took part in times of great uncertainty, naming and discussing things that loomed but were not directly related to the project were hugely valuable in forming and maintaining the group. This included people sharing real time experiences of and concerns about Covid, getting poorly, and getting vaccinated. These discussions in many ways highlight the divide within the group in terms of those with an ‘academic’ interest in the topic and those for whom discussion was intrinsically personal.

Having keen, engaged and knowledgeable advisory group members helped shape and impact the study more than initially intended. This was hugely positive for the research but did mean the research took longer. When planning and costing takes place before research starts, there is not always the manoeuvrability to adequately implement advisory group advice. We often think of the research in purely linear terms moving through the stages in a way pre-determined by our timetables submitted and approved by funders. Decisions are considered and debated but once made rarely revisited. Whereas for the advisory group returning to themes, experiences, and views was an important aspect of the process. This way of doing research is receiving more attention via the lens of crip-time (a lens which challenges the ableist and normative assumptions about time and the speed at which things ‘should’ be done) and its possible ramifications for doing collaborative research differently in terms of time, organisation and pace [34].

Staniszewska et al. [27] shared concerns about the robustness of co-production in times of crisis and this can equally be applied to advisory groups and other methods of inclusive research which need to build flexibility into their approach. The global pandemic and impacts of lockdown highlighted the importance of people with learning disabilities having access to technology and the internet [2, 35]. Online safety has long been a concern for people with learning disabilities themselves as well as families and carers [36]. We can see this reflected in the *British Journal of Learning Disabilities* where two of the top five most cited articles focus on internet usage both with the terms ‘risks’ in their titles [37, 38]. Some of the self-advocacy experts on our project had acquired equipment and learned internet skills because of the pandemic. While funders may assume equipment is provided to university-based researchers by our employers, we may need to think more broadly about providing appropriate equipment and support to key members of research teams who, like our advisory group, sometimes sit outside of institutional support. Without addressing the digital divide, we risk perpetuating inequalities and pose significant ongoing challenges for promoting diversity in public research as we increasingly rely on digital forms of access to our work [39].

One concern shared by fellow researchers is the role of academics in ‘allowing space’ in the research process. For example, the academics involved in Abell et al. [5] expressed concerns over sharing their opinions which they felt could be (or be perceived to be) talking over the voices of people with learning disabilities. In sharing power and no longer positioning academics as the sole experts in the research process, there are uncomfortable realities for academic researchers to face. Whilst we endeavoured to treat voices equally there remained inherent power inequalities. The agendas for the meetings were at first, largely set by the academic researchers who were also being paid to spend more time working on the project than the self-advocacy experts and who were also the budget holders. In more instances than not, the wishes of the academic researchers prevailed when there was conflict with or pushback from the self-advocacy experts. This undermines any claims that could be made about equality of power between different people on the project. Although it helped to discuss this quite explicitly as a group, e.g. in relation to the terminology of learning disability, this might have been mitigated by the involvement of an independent actor in the team, perhaps a facilitator/facilitating organisation paid to attend to these very issues of equalising power relations.

There are often calls to evaluate the impact and science of inclusive research, a ‘What difference does it make?’, plea. This is interesting because such demands are not routinely placed upon non-inclusive research i.e. what difference does it make that none of the research was carried out with people who were the subject of the research. Hewitt et al. [40] argue:

Whilst conducting inclusive research is important, it is equally important to reflect on and evaluate this work to allow greater impact of future inclusive research. Understanding exactly what has happened in inclusive research projects is essential, both in terms of the scope of inclusive researchers, and evaluating the quality of the research…. That inclusive research is challenging is undisputed, but that it adds value to both the quality of research and for individuals involved in the process is also understood. The challenge is for authors to consistently capture both the impact of this added value within the literature, and the difference it makes to the lives of people with intellectual disabilities.

We suggest that our paper contributes towards this in both describing process, suggesting the ‘what difference did it make?’ question, and referencing the views of people with learning disabilities on the impact of being part of the team. We suggest that detail and transparency of research processes and ways of working are good practice in *all* research. But we would not want to place a greater burden of proof or evidence on inclusive research to show that it had benefited the lives of people with learning disabilities given the very complicated routes to research impact.

Coming to the end of the project was difficult. At the time of writing we are still disseminating our findings and presenting at seminars and conferences almost always as a whole team with most of the input coming from self-advocacy experts. We have built relationships on a project which has an end date. We will miss meeting and discussing housing, support, carrier pigeons and drones but want to find ways of continuing to work together and be in touch with each other. The self-advocacy experts certainly wanted to do more with the research:

“We should go further out; we should take it to other places. I think if we go wider out then people might say, ‘this is a problem we’ll get our heads together’ and go to the MP’s or House of Lords and say this is what we found, and they could do a proposal on it.”

“Very sad... it’s a shame we can’t extend it further.”

“Worried that by ending the project the things we have learned will be lost - people will forget what we’ve done.”

Whilst we all want to continue with researching together in various ways, and the academic researchers and collaborators have ongoing involvement in other projects, the path for inclusion of some of the self-advocacy experts is less clear. For us, this is a significant systemic limitation. In bringing public involvement into research we risk building research capacity only to drop this when funding runs out. While we are involved in attempting to create something more sustainable, this is difficult in a research environment that can work against building long-term relationships, a phenomenon described by Ní Shé et al. [41] as “fund and forget.”

## Conclusions

We do not make a grand claim about coproduction in our study. Our study was not a ‘power share’ [27]. Although the overall focus arose out of genuinely mutual sets of conversations and concerns, academics involved people with learning disabilities in aspects of the research design too late and were not sufficiently ambitious in the degree of co-working which could and should have happened. Parts of the research were arguably less successful because they lacked sufficient input from people with lived experience. In a scenario in which the research did not have an advisory group, the academic researchers would have done different research. We would still have talked to people with learning disabilities about what it is like to rent and the support available, but we do not think it would have been as rigorous, reflective, or enjoyable. We might also have missed people who might have been put off by a research project without active involvement from people with learning disabilities.

In reflecting back on our work together we would make the following suggestions (which mostly reinforce points made by the founding voices of inclusive research [7, 8]) to others interested in working in this way, and to wider bodies central to the organisation and delivery of research:

- Work collaboratively from the start. Enter into dialogue about doing research that matters to people who are the focus of the research. Then have conversations about how to work together, who wants to do what, how everybody should be paid, how to ensure that there is equanimity.
- Work out together as a team what model of co-working is going to be adopted. Try and name it truthfully on the continuum of inclusive research. If the team has the scope and the confidence to change that model as the research goes along, for example, if like our project, some people want more input or power, then address that as transparently and openly as possible.
- Consider the benefits of having an external or independent person or organisation involved in the study—perhaps as a meeting facilitator or chair—whose job is to bring issues of power, disagreement and tension out into the open and move towards transparent decision making.
- Attempt a two-way dialogue with your ethics committee about how *they* plan to make *their* processes more accessible and inclusive. Ask them to send their questions in advance in plain English so that your research team can think about them together in advance.
- If there needs to be a ‘flex’ on university rules and regulations in order to be in good relationship with partners, it is important that senior members of the research team take ownership and responsibility for these decisions. We recognise that we are sometimes writing rather opaquely about this issue for reasons which we hope readers will understand. Continue to lobby universities to really understand the full extent of their disempowering and often ableist practices.
- Take time to enjoy the relationship and friendship building that most everybody wants as part of team work on a long’ish research project. Rushing or missing this bit out probably means you won’t work as well together. Be aware of the reciprocity and mutual support going on in the team. The learning will almost certainly work both ways.
- Be sensitive to the many and varied identities that people with learning disabilities hold (just as their non-disabled peers do) and think about how to ensure that you work with and recruit research participants from a range of protected characteristics.
- Be sensitive to the fact that a lot of issues will absolutely not be ‘academic’ to people with lived experience. Decisions made about the research can feel personal. Hearing about exclusion or discrimination will resonate with people’s own pasts and may be difficult to listen to.
- Document the process of working together as you go along so that you can, collaboratively, reflect upon and share details of the strengths, limitations and innovation in your research.

Working together brought joy, challenges and ultimately improved the research in our view. The group were able to act as checks and balances on the academic researchers, helped to appraise the limitations of the research and question the focus of the study. The self-advocacy experts highlighted the gaps in the study’s approach and encouraged the team to think more deeply about those excluded from taking part. They also had views on things that had worked well and things that could be done differently if we all ever worked together again:

“You think about it and get the ideas and then come to us. It would be nice to actually ask me where to go to and what to talk about, come up with an idea together. We could plan it together and all be treated as one.”

“Instead of you doing the work, get some of us involved in the work with you. Work as a team altogether instead of you three doing it all together or working one to one with each other to do things like the phone calls (interviews). Because you don’t have a lot of time to do things and we could help. I know you’ve done very brilliant work with us.”

“You’ve done a good job – a fantastic job including us. I’m glad we’ve had the opportunity to say these things. It’s given us a voice to say how we feel about things. Sometimes we don’t have that opportunity. Professionals talk to you and look down at you all the time, but you don’t.”

We offer these reflections in a spirit of transparency and because we have benefited from those authors who have shared their work and approaches in the past.

## Data Availability

Not applicable.
